# A review of the application of in-vivo confocal microscopy on conjunctival diseases

**DOI:** 10.1186/s40662-024-00409-x

**Published:** 2024-11-01

**Authors:** Mingyi Yu, Chang Liu, Jodhbir S. Mehta, Yu-Chi Liu

**Affiliations:** 1 Tissue Engineering and Cell Therapy Group, Singapore Eye Research Institute, Singapore, Singapore; 2 Department of Cornea and External Eye Disease, Singapore National Eye Centre, The Academia, 20 College Road, Discovery Tower, Singapore, S169856 Singapore; 3 Duke-NUS Medical School, Ophthalmology and Visual Sciences Academic Clinical Program, Singapore, Singapore

**Keywords:** In-vivo confocal microscopy, Conjunctiva, Conjunctival disease, Ocular surface

## Abstract

Over the past few decades, the expanded applications of in-vivo confocal microscopy (IVCM) have greatly enhanced the knowledge of a variety of conjunctival diseases. IVCM allows non-invasively detailed observation of tarsal, palpebral and bulbar conjunctiva, from the superficial to the substantia propria at the cellular level. IVCM has been shown as a powerful tool for the assessment of morphological changes in both physiological and pathological conditions. High-resolution images of different cellular phenotypes, together with quantifiable results, open new insights into understanding the mechanisms of conjunctival diseases, as well as provide valuable and longitudinal information for the diagnosis and therapeutic evaluation. This review aims to provide an overview of the current knowledge on the applications of IVCM on conjunctival disorders, including aging changes, dry eye-related morphological changes, glaucoma and glaucoma surgery-related morphological changes, conjunctival neoplasm, pterygium, allergic conjunctivitis, trachomatous scarring, and the conjunctiva-associated lymphoid tissue (CALT) changes. In this review, we highlight the key findings of previous studies and discusses the current limitations and challenges of IVCM in assessing the structural characteristics of the conjunctiva. Furthermore, we consider possible future directions for unlocking the full potential of IVCM applications. The insights presented here will contribute to a more comprehensive understanding of the applications of IVCM in conjunctival diseases.

## Background

In-vivo confocal microscopy (IVCM) allows in-vivo evaluation of ocular surface and provides information on the morphology features at the cellular level [1, 2]. Using laser light at a wavelength of 670 nm, well-contrast and high-quality images can be obtained [3]. The enface monochrome images at a magnification of approximately 600 times, covering a field of 0.16 mm^2^, with transversal optical resolution of 1 µm and longitudinal optical resolution of 4 µm [4]. Over the past three decades, it has been well-recognized that many ocular and systemic diseases are associated with varying degrees of conjunctival alterations, which can significantly impact the quality of daily life of those affected [5]. However, a significant proportion of substructural changes of the conjunctiva are overlooked because they cannot be observed with a slit-lamp biomicroscope. In addition, sampling of the conjunctiva by traditional histopathological methods, such as impression cytology, can cause unavoidable irritation. Repeated conjunctival sampling further exacerbates the vulnerability of the ocular surface. IVCM overcomes these disadvantages of traditional histopathological examination. It demonstrates objective evidence of structural changes and provides quantifiable data to help clinicians grade the disease severity and monitor disease progression [6]. It also allows for assessing therapeutic efficacy [7] or early detecting the side effects of drugs or surgeries [8]. Several types of confocal microscopes with different principles have been introduced for use in ophthalmology [4], including the tandem scanning confocal microscope, slit scanning confocal microscope, and laser scanning confocal microscope. Tandem scanning confocal microscope was based on the modified Nipkow spinning disc technology, which used a metal disc with multiple pinholes of 30 µm, allowing for true-color, real-time imaging. However, the system had limited light throughput, resulting in relatively low image quality and contrast [9]. The system is no longer commercially available. Slit scanning confocal microscope allows improved light output and faster acquisition time, but at the cost of axial resolution that ranged from 8 to 25 µm, in comparison to 9 to 12 µm in tandem scanning confocal microscope [10]. The laser scanning confocal microscope is currently the most advanced commercially available design. It offers a magnification of 800 times, providing a greater contrast than tandem or slit scanning confocal microscope with a lateral resolution of 1 µm and an axial resolution of 4 µm. Current laser scanning confocal microscopes used in clinical settings include the Heidelberg Retinal Tomograph (HRT), which will be the focus in our review. In addition, all IVCM examinations in this review were performed using the Heidelberg Retinal Tomograph 3 (HRT 3) Rostock Cornea Module (Heidelberg Engineering GmbH, Heidelberg, Germany).

In this review, we summarize and discuss current knowledge about the applications of IVCM on conjunctival diseases, covering conjunctival aging changes, dry eye disease, glaucoma and glaucoma treatments, conjunctival neoplasms, pterygium, allergic conjunctivitis, trachoma and trachomatous scarring, and conjunctiva-associated lymphoid tissue (CALT) changes in ocular diseases.

### Normal IVCM characteristics of the conjunctiva

**Fig. 1:**
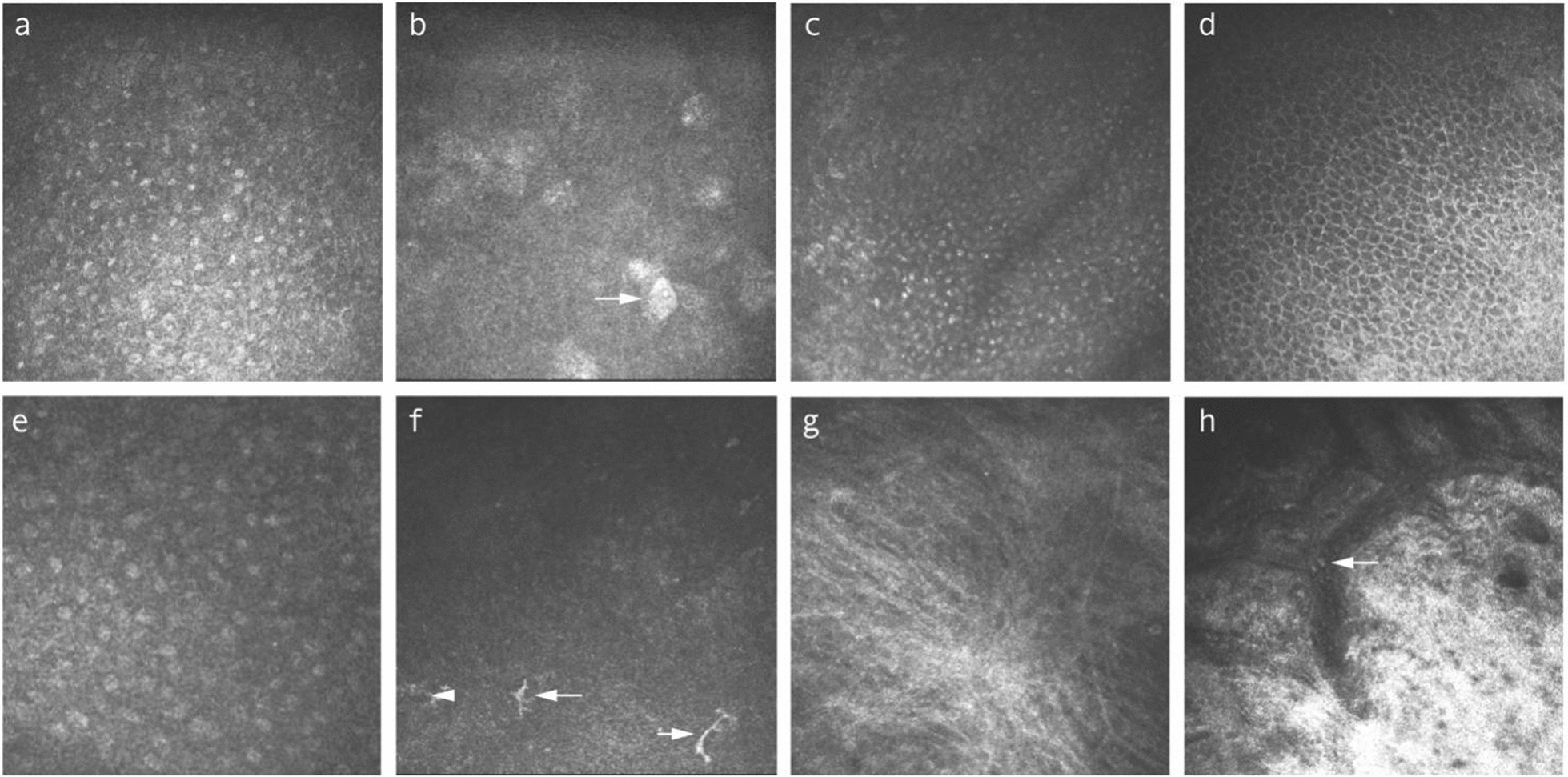
Representative in-vivo confocal microscopy (IVCM) images from normal human conjunctiva. **a** Superficial epithelial cells: large irregular-shaped cells with prominent oval-shaped nuclei. **b** Superficial epithelial cells with hyper-reflective desquamation (arrow). **c** Intermediate epithelial cells manifested as tightly arranged cells with punctate and hyper-reflective nuclei. **d** Basal epithelial cells: polygonal shaped cells with hyper-reflective cell borders and the small bright nuclei visible or not. **e** Goblet cells: large hyper-reflective, ovoid cells of uniform brightness with hypo-reflectivity nuclei. **f** Dendritic cells: hyper-reflective cell bodies, with long dendrites (arrows) or lacking dendrites (arrowhead). **g** Conjunctival lamina propria: a network of hyper-reflective overlapping fibers. **h** Conjunctival lamina propria: blood vessels with hyper-reflective immune cells (arrows)

IVCM allows clear visualization of the conjunctiva from the superficial layer to the conjunctival substantia propria at a depth of approximately 130 µm [11]. Superficial epithelial cells of conjunctiva appear as 10–15 μm sized irregularly-shaped cells, with oval-shaped prominent nuclei, and sometimes hyperreflective desquamation. Intermediate epithelial cells are tightly-arranged cells with punctate, hyperreflective nuclei. Basal epithelial cells have a polygonal shape with hyperreflective cell borders, and the small bright nuclei are visible or absent (Fig. 1a–d). Goblet cells (GCs) are as highly hyperreflective, ovoid cells of uniform brightness with low reflectivity nuclei, typically two to three times larger than the surrounding epithelial cells (Fig. 1e). Dendritic cells (DCs) are typically observed as hyperreflective bodies with dendritic processes distributed in conjunctival epithelium (Fig. 1f). Non-typical morphologies of DCs such as lacking dendrites, long dendrites, or a wire-mesh pattern with long intertwined dendrites may be observed [11, 12]. The conjunctival microcysts can be seen in the intermediate epithelial layer, basal epithelial layer, or subepithelial sites. The microcysts are giant, round, or oval-shaped structures, commonly containing hyperreflective contents and surrounded by hyporeflective rings. With different scanning planes, they can manifest as small granular, highly reflective structures or even vacuoles. The conjunctival lamina propria is located beneath the basement membrane and consists of a complex network of hyperreflective overlapping fibers, and blood vessels with rich immune cells (Fig. 1g, h). The CALT diffuse lymphoid layer is composed of scattered or clustered distributed hyperreflective lymphocytes. CALT lymphoid follicles are well-defined, round-shaped structures that host hyperreflective lymphocytes within a collagenous network [13].

### Aging

Aging is a systemic process, and eyes are no exception to age-related changes. Several ocular diseases are age-related, such as cataract [14], age-related macular degeneration, corneal nerve health [15], and conjunctiva-related disorders such as dry eye and conjunctivochalasis, which commonly coexist in the elderly [16, 17]. IVCM helps to better understand conjunctival changes with age.

Previous studies have reported a decreasing trend in the circularity, size and density of the conjunctival epithelial cells in age-matched healthy individuals although there were no significant differences [11, 15]. However, electron microscopy revealed more pronounced morphological differences, showing a flatter and more elongated shape of the epithelial cells in people over 80 years old compared with the homogeneous polygonal shape of the superficial cells in people aged 50 to 79 years [18]. This may be due to differences in the sensitivity of various methods to detect the ultrastructural structures. Electron microscopy reveals changes in the intercellular space, which was thought to change with age [18].

GCs cluster or scatter throughout the superficial layers of the conjunctival epithelium on IVCM scans. It is generally accepted that the density of GCs continues to increase in early childhood and then remains at a stable level [19] in the healthy population. GCs dysfunction is associated with several age-related ocular surface diseases. However, debate continues about the relationship between GCs density and aging. Zhu et al. [11] evaluated the GCs in the bulbar conjunctiva in different age groups and reported no differences in cell morphology or density among different age groups. Therefore, they hold the view that the changes in GCs were not age-dependent, which is in agreement with the findings of a study on conjunctival biopsy [20]. Nevertheless, this is contrary to the studies that showed a significant decrease in GCs density of palpebral conjunctiva in the aged population [18]. These findings suggest that changes in GCs density may be unsynchronized or independent between bulbar and palpebral conjunctiva with aging, and it has led to research for imaging markers of conjunctival aging on IVCM.

**Fig. 2:**
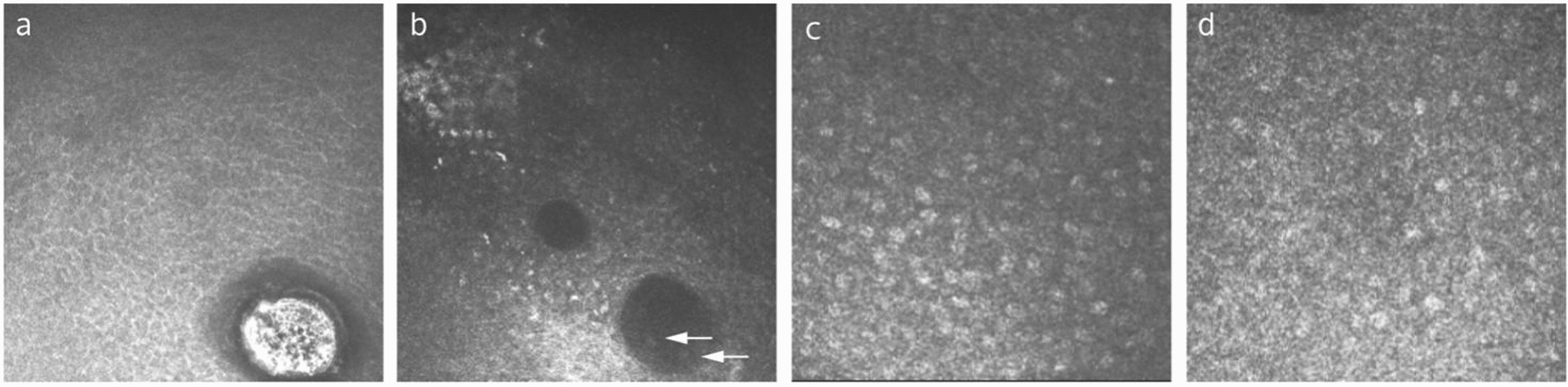
Representative in-vivo confocal microscopy (IVCM) images for young and elderly individuals. **a** Conjunctival microcyst detected in a 64-year-old subject, manifested as a large, oval-shaped structure with hyperreflective contents and surrounded by a hyporeflective ring. **b** Conjunctival microcysts detected in a 71-year-old subject, manifested as dark round vacuoles in various size with or without hyperreflective granules (arrows). **c** Conjunctival epithelium of a 27-year-old subject. Goblet cells are scattered at a high cell density, whereas conjunctival microcysts are absent. **d** Conjunctival epithelium of a 74-year-old subject, showing lower density of goblet cells

Hyaline bodies, characterized by a central or an eccentric granular mass surrounded by a lucent zone, were regarded to be composed of occluded GCs [18]. It was observed in 25% of people over 79 years old [18]. A conjunctival microcyst is another feature that may be age-related (Fig. 2a, b), as previous studies showed that the detection rate of conjunctival microcysts on IVCM was greatly increased with age [11]. Whether conjunctival microcysts are degenerated GCs or normal intermediates that arise during the development and maturation of GCs remains unclear [21]. The age-dependent detection rates of microcysts suggest that the quality of GCs may change with age (Fig. 2c, d). In addition to focusing on GCs density, it may be a more pertinent approach to understand the conjunctival pathological changes with aging by examining changes in the quality of GCs as well as their proper function in mucin secretion.

Several studies have also reported that the density of DCs in the bulbar or the central inferior palpebral decreases with age [20, 22], which is consistent with the results of conjunctival biopsies [23]. Those findings may explain the compromised immune function and increased susceptibility to ocular surface inflammation in elderly. Nevertheless, Wei et al. [24] found no correlation between the density of DCs in the superior palpebral conjunctiva and age. The inconsistent results on the relationship between the DCs density and age could be due to the variation in the scanning area of the conjunctiva.

Previous research has shown that all conjunctival lymphoid structures, including the CALT, undergo age-related changes [25]. Among the IVCM parameters of CALT, the density of lymphocyte, follicular density, and perifollicular lymphocytes density sharply decrease with age following a cubic regression. A significant reduction in follicular area with age, accompanied by a marked increase in follicular reflectivity was also observed [13]. Moreover, the diameter of the fibers network of the conjunctival lamina propria decreased significantly with age [26], which appears to account for the increased conjunctivochalasis in older individuals.

### Dry eye disease

Dry eye disease (DED) is a chronic, multifactorial ocular surface disease with tear film instabilities, hyperosmolarity, and inflammatory damage being major etiological factors [27, 28]. The incidence of DED is increasing worldwide each year and represents a growing burden on those affected by vision and life quality [29]. Aqueous deficient type of DED can be further classified as either Sjögren’s syndrome dry eye (SSDE) or non-Sjögren’s syndrome dry eye (NSSDE) [30]. Primary Sjögren's syndrome is a complex autoimmune disorder with multisystemic effects. It is characterized by lacrimal hyposecretion and permanent inflammation of the ocular surface, leading to severe tear deficiency and gradual epithelial damage on ocular surface [31, 32]. Sustained exposure to hypertonicity and inflammatory stimuli of the ocular surface has been shown to cause the loss of GCs [33]. Conventional cytological examinations of the conjunctiva, such as vital staining, impression cytology, and brush cytology, are important in assessing the density of conjunctival cells and inflammatory infiltrate in DED. Although impression cytology and rose bengal staining are the most sensitive and specific methods of detection [34, 35], frequent impression cytology sampling exacerbates ocular surface vulnerability in SSDE patients. Hong et al. [36] characterized the morphology and density of GCs in SSDE patients under IVCM and confirmed the observations were consistent with those obtained by impression cytology, suggesting that IVCM is a viable alternative to impression cytology in such cases. In addition, the assessment of mid-epithelial or subepithelial inflammatory infiltrates can be difficult with impression cytology. Therefore, IVCM is an effective alternative to assess ocular surface structures at multiple sublayers.

**Fig. 3:**
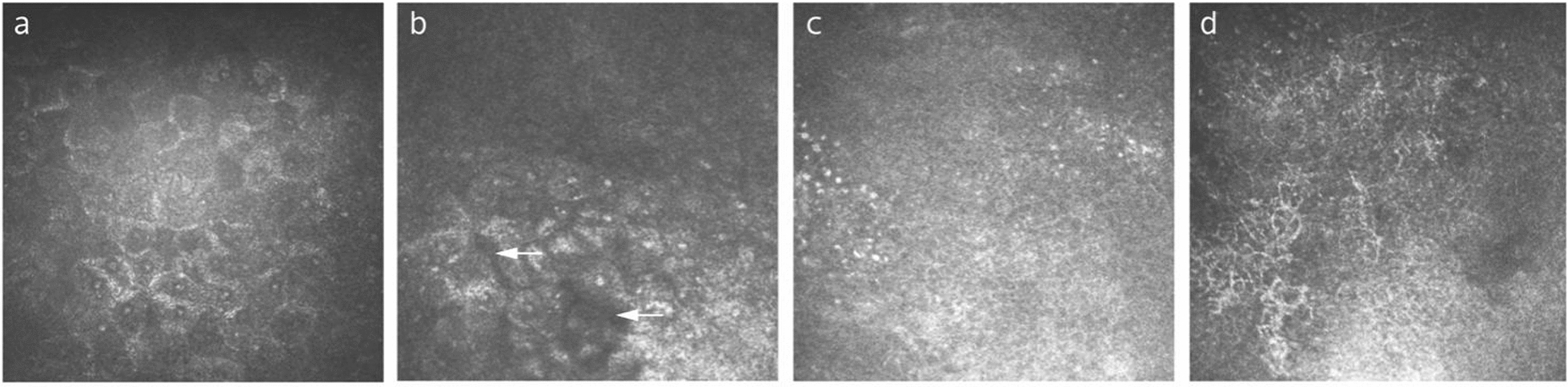
Representative in-vivo confocal microscopy (IVCM) images from a 67-year-old female with non-Sjögren’s syndrome dry eye. **a** Enlargement of conjunctival epithelial cells with nuclear pyknosis. **b** The conjunctival epithelial layer with arrows shows the area of epithelial cells dropping out. **c** Polymorphic inflammatory cells infiltration within the conjunctival epithelium. **d** Wire netting distributed dendritic cells with long dendrites

IVCM examination of SSDE patients showed expanded conjunctival epithelial cells with nuclear pyknosis, decreased nucleocytoplasmic ratio, and even epithelial cell loss (Fig. 3a, b) [37]. Several studies have shown reduced cell density of the bulbar conjunctival epithelium via IVCM in both SSDE and NSSDE patients than normal subjects [37–39]. Both SSDE and NSSDE patients revealed lower basal epithelial cell densities in the bulbar and tarsal conjunctiva compared to controls [40]. This contradicts with another study where the authors reported higher density of corneal basal epithelial cells and anterior stromal cells in the dry eye group compared to the controls [39, 41]. The conflicting findings may result from the fact that DED disrupts the cell renewal but promotes the cell repairment at the same time. Therefore, the apoptosis-proliferation status is very dynamic in corneal and conjunctival epithelial cells. Furthermore, Tais et al. [39] identified a significantly lower conjunctival epithelium cell density in SSDE than in NSSDE. In contrast, Villani et al. [42] detected a higher density of inferior tarsal conjunctival epithelial cells in SS patients. Differences in the site of examination may account for this discrepancy. Yet currently, there are no proven specific IVCM signs to differentiate SSDE from NSSDE.

Inflammatory cell infiltration in the conjunctival epithelium of dry eye patients is observed on IVCM scans. The three major types of inflammatory cells are polymorphs, dendritic cells, and lymphocytes [38]. The density of inflammation cells was higher in DED patients than in controls (Fig. 3c, d), and the SSDE group presented a higher level than NSSDE. Notably, the inflammatory cell density was linked to several dry eye clinical parameters. There was a significant positive correlation between the inflammatory cell density and vital staining scores as well as a significant negative correlation between the inflammatory cell density and the Schirmer test scores and tear breakup time [39]. Hence, inflammatory cell density may provide value in assessing the severity of DED and serve as a potential indicator of treatment efficacy.

Another promising feature of IVCM for the assessment of DED severity could be the conjunctival epithelial microcyst density. Studies on SSDE have demonstrated a significantly higher density of conjunctival microcysts than in the normal subjects although there appears to be no statistically significant difference between SSDE and NSSDE [39].

#### Glaucoma and glaucoma surgery

Glaucoma affects more than 70 million people worldwide and this number is expected to rise to 112 million by the year 2040 [43]. The reduction of intraocular pressure (IOP) with specific modalities including medications, laser therapy, and a variety of surgical interventions [44] has proven to be the most effective management for glaucoma. In addition to monitoring IOP and optic nerve damage, it is becoming increasingly important to monitor the health of the ocular surface. IVCM provides a perspective to elaborate glaucoma-associated conjunctival changes, non-invasively and quantifiably. By providing more morphological details, IVCM also assists in understanding the aqueous humor (AH) drainage pathway [45–47].

#### Untreated ocular hypertension/glaucoma

Ciancaglini et al. [45] examined the bulbar conjunctiva using IVCM and reported microcysts in the conjunctival epithelial layer in both untreated ocular hypertension patients and untreated primary open-angle glaucoma (POAG) patients. These microcysts appeared as round or oval hyporeflective extracellular structures between 10 and 200 µm in size [45]. Similarly, microcysts have also been observed in untreated low-tension glaucoma patients with no significant differences in microcysts mean density (MMD) and microcysts mean area (MMA) as compared to POAG [48]. Nevertheless, the presence of microcysts cannot be used as a distinct feature of glaucoma because microcysts have also been detected in normal subjects without morphological differences [26, 49]. Agnifili et al. [48] showed that microcysts in normal eyes represent the final stage of physiological uveo-scleral AH outflow. The same group also found that although microcysts showed significantly higher MMD and MMA in POAG and low-tension glaucoma patients than in normal subjects, there was no correlation between microcyst parameters and IOP levels [48]. Therefore, it has been proposed that microcyst formation may be activated by hyperbaric ocular conditions at early stages of the disease but hardly changes markedly in response to IOP-induced mechanical forces [45].

#### Medically treated glaucoma

Topical monotherapy is the first-line option for glaucoma. However, over half of the patients require more than two topical medications to keep their IOP within a manageable range [50]. The use of anti-glaucoma medications leads to gradual eye discomfort as well as tear film instability [51]. The conjunctiva undergoes various tissue modifications, and such changes are associated with several factors, including the preserving agents, duration of medication, and combination of medications [52, 53]. Benzalkonium chloride (BAK) is the most common preservative in topical anti-glaucoma medications, which is responsible for most of the ocular surface side effects during long-term therapy [54]. It has been reported that the GCs density, as determined by IVCM, was significantly lower in glaucoma patients treated with BAK-preserved eye drops than in those treated with preserved-free eye drops and controls. Moreover, the BAK-preserved group showed a higher grade of epithelium irregularity and worse ocular surface parameters, such as Schirmer I test and tear breakup time [53, 55].

The conjunctiva can be affected by long-term (more than 12 months) topical treatment from epithelium to stroma. On IVCM examinations, conjunctival epithelium present with squamous metaplasia, cellular desquamation and keratinization, as well as loss of GCs [8, 56]. The activated DCs were observed in the epithelial layer and basal membrane of the conjunctiva [8]. Additionally, the use of multiple (more than 2) therapeutics results in greater negative impact on the conjunctiva, especially the loss of GCs [57]. Loss of GCs could result from the inflammatory state and the accumulation of preservatives on the ocular surface, further destabilizing the tear film. The poor quality of tears makes it difficult for the toxic or inflammatory cytokines of the ocular surface to be flushed, which in turn exacerbates the loss of GCs. The IVCM examination assists in monitoring drug-induced conjunctival damage in patients receiving long-term medication, therefore contributing to the management of glaucoma patients.

#### Surgically treated glaucoma

For patients with IOP uncontrolled by topical medications and with progressive visual field loss, surgical treatment is indicated [58]. Trabeculectomy has been the most effective filtering procedure to lower IOP [58]. The surgical technique typically drains AH from the anterior chamber to the subconjunctival space via a new pathway of intrascleral fistula and involves the elevation of the conjunctiva over the scleral flap, known as filtering blebs [59]. However, the long-term prognosis of surgery can be challenging due to filtering scarring or chronic inflammatory response [47]. The filtrating bleb grading systems known as Indiana Bleb Appearance Grading Scale (IBAGS) and Moorfields Bleb Grading System (MBGS) are the gold standard for assessing filtering bleb function using slit-lamp examination [60, 61]. However, these grading systems do not reflect the underlying pathology of poorly filtered outcomes. IVCM provides morphological insight into the structure of filtering blebs and is a valuable complement to slit-lamp examination. Preoperative IVCM examination may be indicative of filtration prognosis. Studies have shown that IOP reduction at 12 months postoperatively was negatively correlated with preoperative conjunctival DCs density and stromal meshwork reflectivity [62] but positively correlated with preoperative GCs density [62, 63], supporting the hypothesis that "GCs facilitate the AH filtration ability as a cytological carrier" [21, 63]. Inflammatory cell infiltration and increased collagen fibers in the conjunctival stroma on IVCM scans suggest a risk of failed filtration surgery [8]. A high stromal meshwork reflectivity, which is also commonly present in medically treated patients, suggests that the fibrotic changes contribute to the scarring of the filtering blebs [64, 65].

**Fig. 4:**
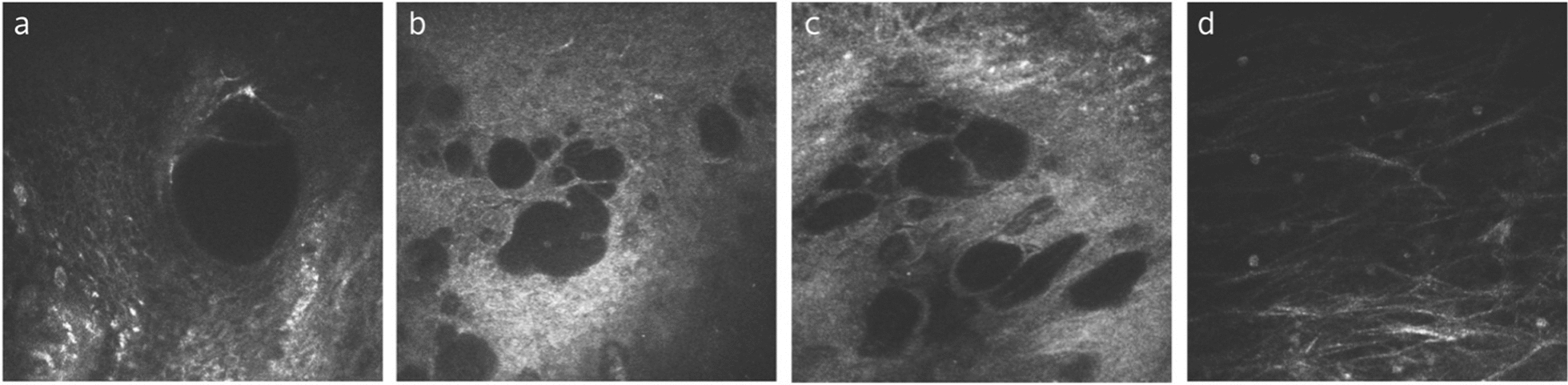
Representative in-vivo confocal microscopy (IVCM) images of functional filtering blebs from a 92-year-old male who underwent trabeculectomy over 10 years ago (intraoperative mitomycin C use unknown) **a**–**c** Examples of large, numerous microcysts in the conjunctival epithelium. **d** Subepithelial connective tissue is extensive and widely spaced

With respect to IVCM features after trabeculectomy, several studies have compared the images in patients with functional and non-functional filtration. A large number of microcysts with irregular distribution over the conjunctiva are present on the functional filtering blebs [63, 66]. Functional filtering blebs are additionally hallmarked by large total cyst area, non-encapsulated, minimal vascularization, and absence of tortuous conjunctival vessels (Fig. 4a–c) [67, 68]. In contrast, non-functional filtering blebs showed limited or absent microcysts, indicating poor AH percolation through the conjunctiva [66]. Functional filtering blebs also had numerous atypical GCs, whereas non-functional filtration blebs presented a significantly lower GCs density [63, 66]. Functional filtering blebs present extensive, widely spaced subepithelial connective tissue (Fig. 4d) while non-functional blebs appear dense [47]. In the conjunctival stroma of filtering blebs, a loosely structured collagenous network is a sign of a well-functioning bleb. On the contrary, a dense and hyperreflective stroma that represents fibrosis is associated with bleb dysfunction. Guthoff et al. further classified the conjunctival stromal features shown by IVCM into four subtypes [46]. They found that the early and late functional blebs were typified by a trabecular or reticular stromal pattern, respectively, whereas early and late non-functional blebs were typified by a compacted or corrugated stromal pattern. Lastly, mitomycin C reduces postoperative subconjunctival fibrosis in trabeculectomy and maintains filtering function [69]. With the use of intraoperative mitomycin C, filtering blebs showed marked increased in MMD and MMA, and a large unencapsulated cystic pattern on IVCM, which was in line with the filtration enhancement [70].

In addition to trabeculectomy, several other procedures are also available, and the associated conjunctival changes on IVCM have been reported. Ultrasonic circular cyclocoagulation is a cyclo-destructive technique that reduces the formation of AH and stimulates the suprachoroidal and uveoscleral outflow pathways [71]. Mastropasqua et al. reported a significant increase in MMD and MMA in patients who underwent ultrasonic circular cyclocoagulation, indicating the increased trans-scleral AH outflow to be a possible mechanism for IOP control [72]. Gold micro shunt implantation is a bleb-less surgical procedure that creates channels between the anterior chamber and suprachoroidal spaces, and thus takes advantage of the natural hydrostatic pressure gradient (1 to 5 mmHg) and leads AH to flow via the choroidal vascular system or the scleral layers, and then the conjunctiva [67]. Mastropasqua et al. examined the characteristics of the suprachoroidal spaces after the shunt implantation and found a greater MMD and MMA in the successful implantation (1/3 reduction in IOP) than in the unsuccessful implantation [73]. However, the extent to which micro shunt implantation improves the filtration is unclear due to a lack of baseline measurements. The Xen 45 Gel Stent, a soft permanent device shunting AH from the anterior chamber to the subconjunctival space, is increasingly being used as a means of minimally invasive glaucoma surgery [74]. The Xen 45 Gel Stent causes limited disturbance to the ocular surface, providing a milder inflammatory response compared to the filtration surgery [75, 76]. Higher MMD with lower subepithelial connective tissue density was reported [77]. Table 1 summarizes the conjunctival changes on IVCM evaluation in different types of glaucoma and glaucoma treatment, and Table 2 provides a concise overview of the shared insights across the literature.

**Table 1:** IVCM findings of conjunctival morphology in glaucoma and glaucoma surgery

Authors	Study population	Treatment	IVCM findings
Agnifili et al. [48]	Low-tension glaucoma, POAG	NA	MMD and MMA were significantly higher in POAG and low-tension glaucoma patients than in normal patients, but showed no significant differences between POAG and low-tension glaucoma patients
Ciancaglini et al. [53], Frezzotti et al. [55]	Ocular hypertension, POAG	Medical treatment (preserved vs. unpreserved)	Significantly lower GCs density, higher irregularity grade of conjunctival epithelium in preserved eye drops treated than in preserved-free eye drops treated patients
Mastropasqua et al. [8]	POAG	Medical treatment (long-term vs. short-term)	Long-term (12 months) topical treatment leads to squamous metaplasia, cellular desquamation, keratinization and loss of GCs in conjunctival epithelium; DCs activation in the epithelium and the basal membrane; inflammatory cell infiltration and increased collagen fibers in the conjunctival stroma
Staso et al. [57]	POAG, pseudo-exfoliative glaucoma, pigmentary glaucoma	Medical treatment (combination therapy vs. monotherapy)	Combination therapy (more than 2 drugs) leads to greater loss of GCs than monotherapy
Mastropasqua et al. [62]	POAG	Trabeculectomy	Preoperative DCs density and stromal meshwork reflectivity negatively correlated with postoperative IOP reduction; preoperative GCs density positively correlated with 12-month IOP reduction
Agnifili et al. [63]	POAG		
Agnifili et al. [63]	POAG	Trabeculectomy	Higher GCs density in functional filtering blebs than in non-functional filtration blebs
Labbe et al. [47]	POAG	Trabeculectomy	Subepithelial connective tissue present extensive and widely spaced in functional filtering blebs, and present dense non-functional blebs
Guthoff et al. [46]	Primary/secondary open angle glaucoma, angle-closure, developmental glaucoma	Trabeculectomy	The early and late functional blebs were trabecular or reticular in stromal pattern, respectively, while early and late non-functional blebs present compacted or corrugated stromal pattern
Ciancaglini et al. [70]	POAG	Trabeculectomy	Marked increased in MMD and MMA, and present large unencapsulated cystic pattern with mitomycin C applied
Mastropasqua et al. [72]	Glaucoma (with history of more than 1 incisional surgeries)	Ultrasonic circular cyclocoagulation	Significant increase of the MMD and MMA
Mastropasqua et al. [73]	Glaucoma (with history of multiple failed incisional surgeries)	Gold micro shunt implantation	Greater MMD and MMA in successful implantation (1/3 reduction in IOP) vs. unsuccessful implantation
Fea et al. [77]	POAG	Xen 45 gel stent implantation	Higher MMD and lower subepithelial connective tissue density

*IVCM *= in-vivo confocal microscopy; *POAG* = primary open-angle glaucoma; *MMD *= microcysts mean density;* MMA *= microcysts mean area; *GCs *= goblet cells;* DCs* = dendritic cells; *IOP *= intraocular pressure

**Table 2:** Summary of IVCM findings of glaucoma and glaucoma surgery

Type of glaucoma and therapy	IVCM findings
Ocular hypertension [53]	• Presence of microcysts in the conjunctival epithelial layer
POAG [48, 57]	• Presence of microcysts in the conjunctival epithelial layer with large MMD and MMA
Post glaucoma surgeries (trabeculectomy/ultrasonic circular cyclocoagulation/gold micro shunt/Xen 45 gel stent treated glaucoma) [47, 63, 72, 73, 77]	• Functional filtering blebs: microcysts present as large number, large area, non-encapsulated, minimal vascularization, without tortuous conjunctival vessels with irregular distributing over the conjunctiva; numerous atypical GCs; extensive, widely spaced subepithelial connective tissue • Non-functional filtering blebs: limited or absent microcyst; less GCs; dense and hyperreflective stroma; • Greater MMD and MMA represent a more successful treatment

*IVCM *= in-vivo confocal microscopy;* POAG* = primary open-angle glaucoma; *MMD* = microcysts mean density;* MMA * =  microcysts mean area;* GCs* = goblet cells

### Conjunctival neoplasm

Conjunctival neoplasms cover a wide range of the disease spectrum and may involve the epithelial layer to the stromal layer. Pigmented conjunctival tumors are the most common types [78], among which the main forms are melanoma, nevus, primary acquired melanosis (PAM), and pigmentary melanosis [79]. Pathological analysis remains the current gold standard as various conjunctival neoplasms have overlapping clinical features and make clinical diagnosis challenging [80]. IVCM is a non-invasive way to visualize the histological features and provide additional information to diagnose and differentiate the conjunctival neoplasms.

The nevi on bulbar conjunctiva show nests or diffuse accumulation of medium-sized hyperreflective cells, as well as multilayered epithelioid cysts within conjunctival stroma [81]. Lacrimal caruncle conjunctival nevi account for 8.2% of conjunctival pigmented lesions and are characterized by cells with clear borders, hyperreflective periphery, and hyporeflective center, grouped in nests with irregular borders, and vessels surrounding the nevus [82]. PAM without atypia showed hyperreflective granules in 67% of lesions and small dendritic cells (< 20 μm) in all lesions restricted to the basal epithelium, while PAM with atypia presented networks of large (> 20 μm) dendritic cells and hyperreflective cells, granules and patches throughout the epithelium [83]. Large atypical hyperreflective cells with prominent nuclei were seen in malignant conjunctival melanomas with 89% sensitivity and 100% specificity [83, 84]. Moreover, there is a strong correlation between IVCM and histological findings. On IVCM, a cystic structure was observed with the nested distribution of hyper- and hyporeflective monomorphic cells, which is consistent with the epithelial pseudocysts and pigmented and non-pigmented nevus cells, as demonstrated by histological examinations. PAM without atypia showed a hybrid of hyperreflective cells and DCs confined to the basal epithelium on IVCM. This finding corresponds to the pigmented hyperplasia observed in pathology, which was also limited to the basal epithelium [83, 84]. Therefore, IVCM may have value in the differentiation of pigmented conjunctival lesions before biopsy. An accurate differentiation is crucial in clinical practice for proper management and prognosis. Secondary conjunctival pigmentation can be associated with various systemic conditions, including Addison’s disease [85], pregnancy [86], and other states with hormonal changes. Additionally, certain medications like calcium channel blockers [87], as well as exposure to arsenic [88], Thorazine toxicity [89], radiation [90], or foreign materials/implants [91], may cause conjunctival pigmentation. Currently, the differential diagnosis mentioned above mainly depends on histopathological evidence, with very few reports on IVCM features. This might be one of the directions for future research.

Ocular surface squamous neoplasia (OSSN) is the most common malignant tumor of the ocular surface [92]. The clinical features of OSSN may overlap with those of benign lesions, as no signs are specific for OSSN. IVCM of conjunctival OSSN reveals hyperreflective cells of various sizes, prominent basal cell nuclei, and amorphous hyperreflective features (possibly keratinous material) [92, 93]. Nevertheless, the sensitivity and specificity of IVCM to distinguish OSSN from benign conjunctival lesions were only 38.5% and 66.7%, respectively [93]. Even so, it may still help clinicians determine treatment response and disease relapse.

Conjunctival lymphomas are mainly composed of extranodal marginal zone B-cell lymphomas of the mucosa-associated lymphoid tissue (MALT) [94]. On IVCM, the lesion shows polygonal cells without hyperreflective borders or visible nuclei. Small hyperreflective cells may also be observed nested within cystic hyporeflective spaces or between stromal collagen fibers [95].

There have been reports of rare conjunctival tumors with IVCM features. Lam et al. [96] reported a patient with multiple endocrine neoplasia type 2B (MEN 2B) syndromes with conjunctival neuroma due to an axon and Schwann cell abundance [97]. IVCM of lesions showed large, thick, disorganized nerve bundles with prominent loops and branching that correspond to the subconjunctival nerves known as conjunctival neuromas. The walls of the neuroma were hyporeflective and numerous individual hyperreflective elementary nerves were seen within it [96].

In conclusion, IVCM is a rapid and non-invasive way for detailed and longitudinal observation of conjunctival neoplasms in a real-time manner, providing an effective adjunct to histopathology. Numerous studies have described the IVCM characteristics of various conjunctival neoplasms (Table 3). Even though histopathology remains the gold standard for diagnosis, IVCM has improved the understanding of the natural history of various conjunctival neoplasms. It should be noted that IVCM has limitations in the detection of conjunctival neoplasms. These include limited tissue translucency, which may cause images of deep or poorly translucent tissue to be blurred. In addition, IVCM cannot differentiate between cells with similar refraction index and morphology, such as activated Langerhans cells and dendritic melanocytes [82].

**Table 3:** IVCM features of conjunctival neoplasms

Authors	Type of neoplasms	IVCM findings
Cinotti et al. [81]	Conjunctival nevi	Nests or diffuse accumulation of medium-sized hyperreflective cells; multilayered epithelioid cysts within conjunctival stroma
Cai et al. [82]	Nevus on the caruncle conjunctiva	Cells with clear borders, hyperreflective periphery, and hyporeflective center, grouped in nests with irregular borders; vessels surrounding the nevus
Messmer et al. [83]	PAM without atypia	Hyperreflective granules in 67% of lesions; small (< 20 μm) dendritic cells restricted to the basal epithelium in 100% of lesions
	PAM with atypia	Large (> 20 μm) dendritic cells networks; hyperreflective cells, granules, patches throughout the epithelium
Ronin et al. [84]	Conjunctival malignant melanomas	Large atypical hyperreflective cells, prominent nuclei
Pichierri et al. [95]	Conjunctival lymphomas	Polygonal cells without hyperreflective borders or visible nuclei; small hyperreflective cells within hyporeflective spaces or between stromal collagen fibers
Nguena et al. [93]	Ocular surface squamous neoplasia	Hyperreflective cells of variable size; prominent basal cell nuclei; amorphous hyperreflective features (possibly keratinized material)
Lam et al. [96]	Conjunctival neuroma	Large, thick, disorganized nerve bundles with prominent loops and branching; hyporeflective walls of the neuroma; numerous individual hyperreflective elementary nerves within the neuroma

*IVCM *= in-vivo confocal microscopy; * PAM *= primary acquired melanosis

### Pterygium

**Fig. 5:**
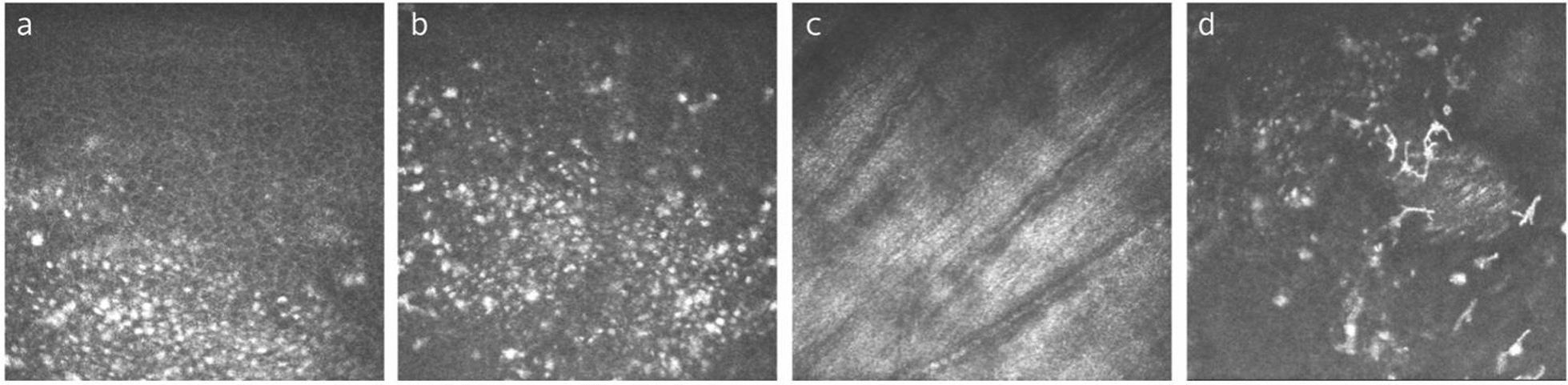
Representative in-vivo confocal microscopy (IVCM) images from a 64-year-old female with primary pterygium in the nasal conjunctiva. **a** Hyperreflective epithelial cells of the pterygium invaded the corneal basal epithelium at the irregular pterygium-cornea junction. **b** Infiltration of hyperreflective inflammatory cells distributed throughout the pterygium stroma. **c** Abundant blood vessels in the pterygium stroma. **d** Abundant dendritic cells distributed throughout the pterygium body as a branching pattern

Pterygium is the growth of fleshy fibrovascular tissue from the bulbar conjunctiva on the cornea. IVCM provides information of morphological changes over time and visualization of the response to topical mitomycin C application in pterygium surgery. The epithelium of patients with pterygium consists of highly reflective cells (Fig. 5a) [98]. Evidence of pterygium in the conjunctiva includes the presence of inflammatory cells in the pterygium epithelium and stroma, and abundant blood vessels (Fig. 5b, c) [98]. The DC density is significantly higher in the body of pterygium than in the nasal bulbar conjunctiva of the healthy with a typical branching pattern (Fig. 5d) [99]. The epithelial or sub-epithelial layer of the pterygium consists of microcysts with hyperreflective content, while the stroma of the pterygium is fibrovascular with sparse inflammatory cells infiltrated [99, 100]. GCs were observed in the pterygium epithelium, however, the cell density decreased after the application of mitomycin C during surgery without returning to normal levels 4 weeks postoperatively [99, 101]. This suggests that even though mitomycin C reduces pterygium recurrence by inhibiting cell proliferation, it may disrupt mucin production, which increases the risk of dry eye. Previous research has identified features in pterygiums that mimic neoplasms, including unlimited growth and local tissue invasion [102]. Immunohistochemical analysis of primary pterygium tissue has demonstrated significant expression of survivin, which is highly upregulated in almost all types of human malignancy [103]. However, currently, there are no IVCM [104] or other non-invasive approaches available that can accurately differentiate between the features of cancerous growths within pterygium lesions.

### Allergic conjunctivitis and vernal keratoconjunctivitis

**Fig. 6:**
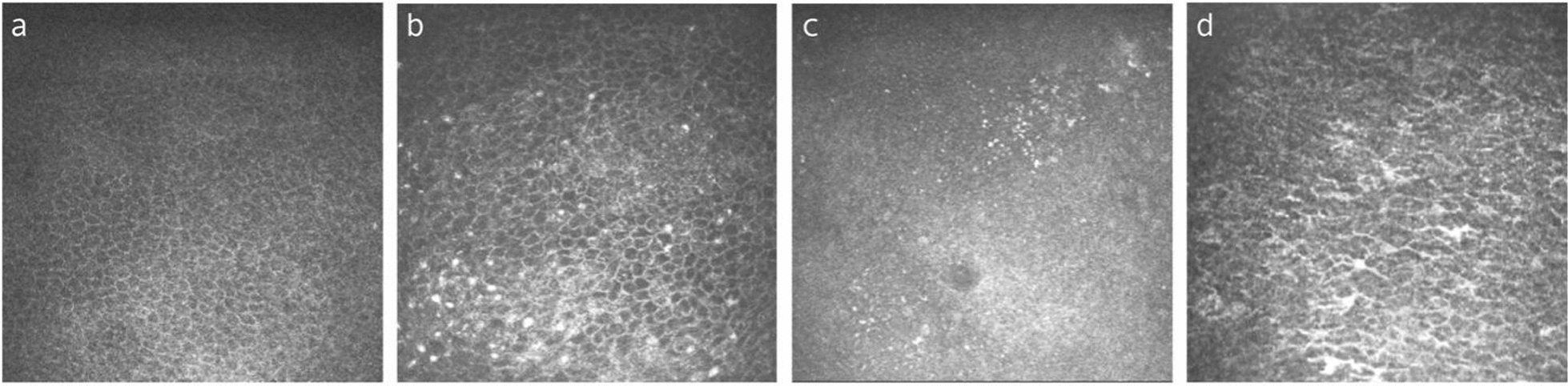
Representative in-vivo confocal microscopy (IVCM) images of the bulbar conjunctiva from a normal subject and a 33-year-old female with atopic conjunctivitis. **a** Normal subject, notice the absence of inflammatory cells and dendritic cells in the basal membrane of the conjunctiva. **b**–**d** Allergic conjunctivitis patient with numerous infiltrated inflammatory cells and scattered dendritic cells visible in the basal membrane of the conjunctiva

Allergic conjunctival diseases are a group of conjunctival inflammatory conditions that are triggered by various types of hypersensitive reactions [105]. Allergic conjunctival diseases are classified into four main types, including allergic conjunctivitis, atopic keratoconjunctivitis (AKC), vernal keratoconjunctivitis (VKC), and giant papillary conjunctivitis, based on the presence of proliferative changes, complicated atopic dermatitis, and mechanical foreign body irritation [105]. Inflammation and the release of allergenic mediators at the ocular surface are major contributors to the histological changes [106]. During the allergic immune response, DCs capture, process, and deliver antigens [107]. DCs accumulate in clusters, with a wire-netting pattern observed in 38% of the allergic patients, leading to significantly higher DCs density compared to healthy controls (Fig. 6) [108]. Future studies should focus on DC morphology and correlate that with the level of markers of conjunctival DCs, to determine whether DCs features could potentially be used as a biomarker of the disease severity.

**Fig. 7:**
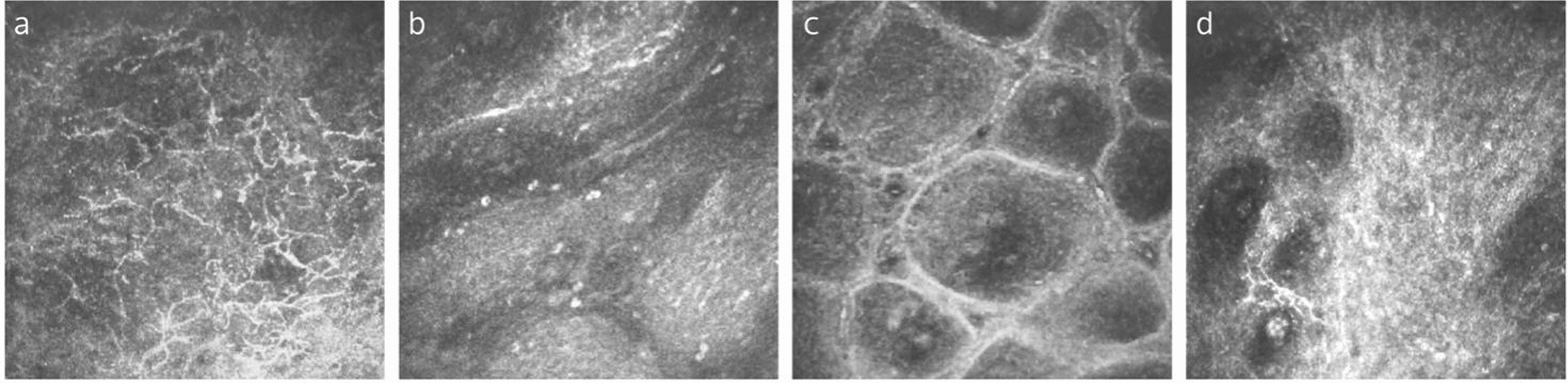
Representative in-vivo confocal microscopy (IVCM) images from a 19-year-old male with vernal keratoconjunctivitis. **a** Numerous dendritic cells infiltrated the tarsal conjunctiva. **b** and **c** The papillae structure in the upper tarsal conjunctiva with various sizes, infiltrated with inflammatory cells. **d** Proliferation of fibrous tissue in the deep stroma of the tarsal conjunctiva

VKC is a chronic, bilateral allergic disease that typically affects young males [109]. The hallmark of VKC is papillary hyperplasia affecting the upper tarsal and limbal areas; accordingly, VKC is classified as tarsal form, bulbar form, and mixed form [110]. IVCM examinations showed that both tarsal and bulbar conjunctiva were infiltrated with a greater number of DCs in the conjunctival stroma (Fig. 7a) and inflammatory cells in the conjunctival epithelium when compared to healthy subjects [111]. These findings are in line with histopathological biopsy results [112]. Moreover, DCs density in tarsal conjunctiva was significantly higher in the tarsal and mixed forms of VKC than in the bulbar form, suggesting that the severity of the immune dysfunction is greater in the tarsal and mixed forms [111]. In addition, the papillary structure can be clearly seen in the upper tarsal conjunctiva (Fig. 7b, c), presenting a ring-shaped structure with a hyperreflection at the center and a hyporeflection at the periphery, infiltrated with a number of inflammatory cells and DCs [111]. Giant papillae are considered to be the evidence of fibroblast activation and tissue remodeling in VKC, with the natural progression being scar formation [113]. Le et al. [114] reported a patient with a 14-year history of mixed form VKC, who developed a significant proliferation of fibrous in the deep stroma of the tarsal conjunctiva on IVCM evaluation (Fig. 7d). This finding is consistent with the results which were presented by Leonardi et al. [106], suggesting that conjunctival fibrosis or scarring results from the long-standing chronic allergic states in VKC.

To conclude, IVCM is able to visualize the conjunctival changes in patients with allergic conjunctival diseases, serving as a supplementary tool to biopsy-based histopathology. Currently, there are no longitudinal observations on the IVCM characteristics throughout the course of the disease, and this can now be used as a reference for monitoring the disease process and evaluating treatment response.

### Trachoma and trachomatous scarring

Trachoma is a chronic, scarring keratoconjunctivitis caused by the recurrent infection with Chlamydia trachomatis [115]. Active Chlamydia trachomatis infection typically causes follicular and papillary conjunctivitis, occasionally with corneal pannus, while repeated episodes of infection may result in tarsal conjunctival scarring, trichiasis, and even corneal opacity [116–118]. Trachoma remains the most common infectious cause of blindness globally with approximately 131 million cases each year and rising; it is also responsible for severe visual impairments in about 1.9 million individuals worldwide [119].

Hu et al. [120] described the IVCM features of the tarsal conjunctiva in both active trachoma and trachomatous conjunctival scarring subjects. Follicular and papillary structures in the tarsal conjunctiva are the key features of active trachoma. In addition, multiple black “cystic” spaces or lacunae, indicating areas of tissue edema, were observed in nearly half of the actively infected individuals. Numerous interdigitated distributed DCs with long processes were observed [120]. On the other hand, patients who develop trachomatous conjunctival scarring are typically found to have inflammatory cell infiltration, increased DC density, and connective tissue organization at the subepithelium on IVCM images [121]. The density of inflammatory cells and DCs showed a significantly positive correlation with clinical scarring scores, indicating that the elevated cellularity was strongly associated with trachomatous scarring process and trichiasis formation [121].

A 4-point IVCM grading system was proposed to evaluate conjunctival connective tissue scarring for trachoma and other conjunctival diseases, which demonstrated good agreement with clinical scarring scores. In brief, Grade 0 (normal) refers to homogenous, amorphous appearance with occasional fine, wispy strand; Grade 1 refers to heterogeneous appearance with poorly defined clumps or bands; Grade 2 has clearly defined bands of tissue that constitute 50% of the scan field; while Grade 3 has clearly defined bands of tissue that constitute 50% of the scan field with visible striations [120]. A study further confirmed a strong correlation between the IVCM and histopathological or immunohistochemical gradings [122].

In summary, IVCM demonstrated morphological alterations in the conjunctiva at a cellular level in the cases of trachoma. The IVCM findings, such as increased density of inflammatory cells and DCs, as well as connective tissue organization, have identified major promoters of trachomatous conjunctival scarring with potential targets for controlling the fibrotic process. Moreover, the IVCM-based grading system provides quantitative evidence of monitoring the conjunctival scarring progression.

### CALT changes in ocular diseases

The CALT, together with the lacrimal and lacrimal drainage-associated lymphoid tissue, composes the eye-associated lymphoid tissue [123]. Multiple studies have demonstrated the morphological structure of CALT, which consists of diffuse lymphoid layer, lymphoid follicles, and crypt-associated lymphoid structures [13, 25]. CALT functions as the immunological interface between the ocular surface and the external environment, and is an important component of the immune defense of the eye [124].

In patients with glaucoma, the reticular structures in the follicles in CALT appear to be more reflective compared to those in healthy subjects, indicating the deposition of collagen and lipoid substances [66]. Follicles infiltrated with scattered or clustered lymphocytes also have been observed in patients with medically controlled glaucoma, with these features being more obvious in patients treated with a combination of multiple drugs compared to those on monotherapy [66].

In patients with DED, the CALT presents as numerous hyperreflective lymphocytes infiltrating the diffuse lymphoid layer, follicles, and follicular spaces. As the disease progresses, the number of lymphocytes increases and tends to become more clustered, suggesting a greater dysregulation and activation of CALT [56]. Another study quantified the CALT features of meibomian gland dysfunction patients. Compared with healthy subjects, meibomian gland dysfunction patients showed significantly increased density of diffuse lymphocytes, follicles, and perifollicular lymphocytes, and a greater reflectivity of the central follicles. There was no significant difference in the follicle area between the meibomian gland dysfunction and healthy subjects [125]. Furthermore, there was a positive correlation between diffuse lymphocyte density and telangiectasia, and between central follicle reflection intensity and meibomian gland blockage [125].

**Fig. 8:**
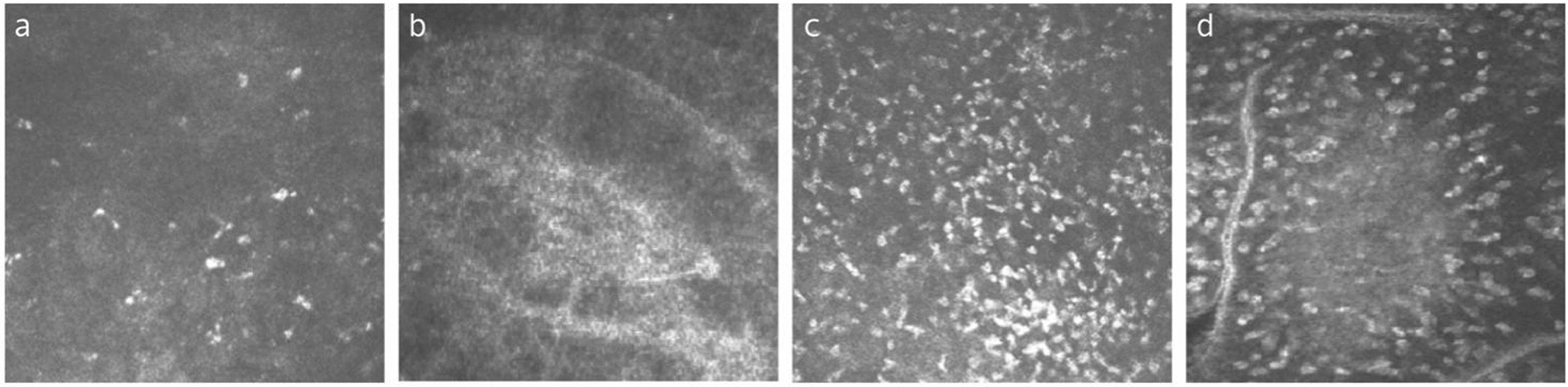
Representative in-vivo confocal microscopy (IVCM) images of conjunctiva-associated lymphoid tissue (CALT) from a normal subject and a 52-year-old male with herpes simplex virus keratitis. **a** The diffuse lymphoid layer of the healthy subject shows a low density of highly reflective lymphocytes. **b** The lymphoid follicles of the healthy subject manifested as round-shaped with a low density of lymphocytes inside. **c** The diffuse lymphoid layer of the infectious keratitis patient presented as an infiltration of numerous hyperreflective lymphocytes. **d** Lymphoid follicles of the infectious keratitis patient showed irregular-shaped follicles, a greater number of parafollicular lymphocytes, and a larger follicular area

Similarly, patients with infectious keratitis showed a significantly higher density of diffuse lymphocytes, follicles, perifollicular lymphocytes, and follicular center reflection, indicating an activated state. Differing from that of meibomian gland dysfunction, the follicular area was significantly higher in keratitis patients than in healthy subjects (Fig. 8). With treatment, the CALT activation gradually subsides, evidenced by the reduction in lymphocyte densities and follicular area. However, no significant differences in CALT parameters, such as the density of diffuse lymphocytes, follicles, perifollicular lymphocytes, follicular area and follicular center reflection intensity, were observed between patients with keratitis due to different types of pathogens [126].

Table 4 summarizes the IVCM findings on CALT in various ocular diseases. A more comprehensive understanding of the progression and regression of the disease can be achieved by the quantitative assessment of the CALT features in combination with the clinical signs. Careful identification of CALT from other rounded structures in tarsal conjunctiva, such as papillae, should not be overlooked. Therefore, immunohistochemistry is warranted to identify specific tissue and cellular phenotypes, where appropriate.

**Table 4:** IVCM features of CALT in various ocular diseases

Authors	Ocular disease	IVCM findings
Mastropasqua et al. [66]	Glaucoma	• Higher reflective reticular structures in follicles in glaucoma patients than in normal subjects • More evident lymphocytic infiltration in follicles with topical combined therapy than with monotherapy
Mastropasqua et al. [56]	Dry eye	• Numerous lymphocytes infiltrate into the diffuse lymphoid layer, follicles, and follicular spaces • Lymphocyte density increases and tend to cluster as dry eye advances
Liu et al. [125]	Meibomian gland dysfunction	• High density of diffuse lymphocytes, follicles, and perifollicular lymphocytes • Great reflectivity of the central follicles • Positive correlation between diffuse lymphocyte density and telangiectasia, and between central follicle reflection intensity and blockage
Liu et al. [126]	Infectious keratitis	• High density of diffuse lymphocytes, follicles, and perifollicular lymphocytes • Large follicular area • Great reflectivity of the central follicles • Reduction of diffuse lymphocyte density, parafollicular lymphocyte density, and follicular area as keratitis improved

*IVCM* = in-vivo confocal microscopy; *CALT * = conjunctiva-associated lymphoid tissue

### Miscellaneous

#### Chemical injury

Exposure of the ocular surface to acidic or alkaline chemicals is known to cause severe damage to the cells and tissues. Le et al. [127] found that patients who suffered from chemical burns showed a marked reduction in the density of GCs in the conjunctiva, which was consistent with the results of the impression cytology examination. GCs deficiency is associated with an increased risk of poor tear film stability and keratoconjunctivitis sicca, the most common long-term complication of chemical burns [128, 129]. Notably, GCs were also detected on the cornea in 15.5% of chemically injured eyes. The ingrowth of GCs onto the cornea was considered a hallmark of limbal stem cell deficiency [130], making IVCM a potentially valuable tool for predicting injury outcome.

#### Superior limbic keratoconjunctivitis

Superior limbic keratoconjunctivitis (SLK) is characterized by inflammation of the upper bulbar conjunctiva, and punctate staining of the superior limbal cornea and adjacent bulbar conjunctiva [131]. Kojima et al. [132] evaluated the superior bulbar conjunctiva in SLK patients. They found an enlargement in cell size and decreased nucleocytoplasmic ratio in conjunctival epithelial cells, along with an increased density of inflammatory cells in the epithelium layer. Notably, IVCM parameters of conjunctival epithelial cells revealed a strong linear correlation with impression cytology examination. The inflammatory cell density in IVCM was also significantly correlated with the rose bengal conjunctival staining scores.

#### Graves’ ophthalmopathy

When the ocular surface is affected by systemic diseases, IVCM is a useful tool for observing the morphological changes resulting from underlying systemic disease activity. In patients with Graves’ ophthalmopathy, the density of epithelial cells in the superior bulbar conjunctiva was lower than that in the healthy subjects. This reduced density was also significantly negatively correlated with patients’ ocular surface disease index scores [133]. Higher DCs density and lower GCs density were also noted [133]. These findings indicate that lagophthalmos and upper lid retraction in Graves’ ophthalmopathy can lead to poor tear-film protection and inflammatory damage of the ocular surface.

#### Chronic graft-versus-host disease

Among individuals who develop chronic graft-versus-host disease (GVHD) after allogeneic hematopoietic stem cell transplantation, 60%–90% have ocular involvement, with dry eye disease being the most common ocular complication [134, 135]. Kheirkhah et al. [136] compared the IVCM features of conjunctival epithelial immune cells between dry eyes associated with or without GVHD using IVCM. They found a higher cell density in both GVHD and non-GVHD associated dry eye than in healthy eyes. However, no significant difference was found between the two dry eye groups. Changes in IVCM characteristics alone may not account for the whole range of abnormalities in GVHD-associated dry eye [136].

#### Sunlight exposure

There has been a growing interest in ocular surface changes associated with sunlight exposure in recent years. Several diseases are associated with excessive ultraviolet exposure, including pinguecula and pterygium [137]. Grupcheva et al. found that the detection rate of conjunctival cysts was significantly higher after summer sunlight exposure compared to baseline. Additionally, the total area of the cysts was 20 times larger after sunlight exposure, with a gradual recovery of the structural changes over time [138]. A strong negative correlation between the use of sun protection products and the number of conjunctival cysts demonstrates the protection effect at the cellular level [138].

#### Amyloidosis

Conjunctival amyloidosis is a common form of ocular involvement in amyloidosis, which is a spectrum of disorders characterized by the deposition of insoluble fibrillar proteins in the extracellular and perivascular space [139]. Under IVCM, the lesions showed hyporeflective deposits in a lobular pattern in the substantia propria and surrounding blood vessels, with no distribution of inflammatory or DCs within or around the lesion. The IVCM findings were consistent with histopathological findings.

#### Retinal surgery

**Fig. 9:**
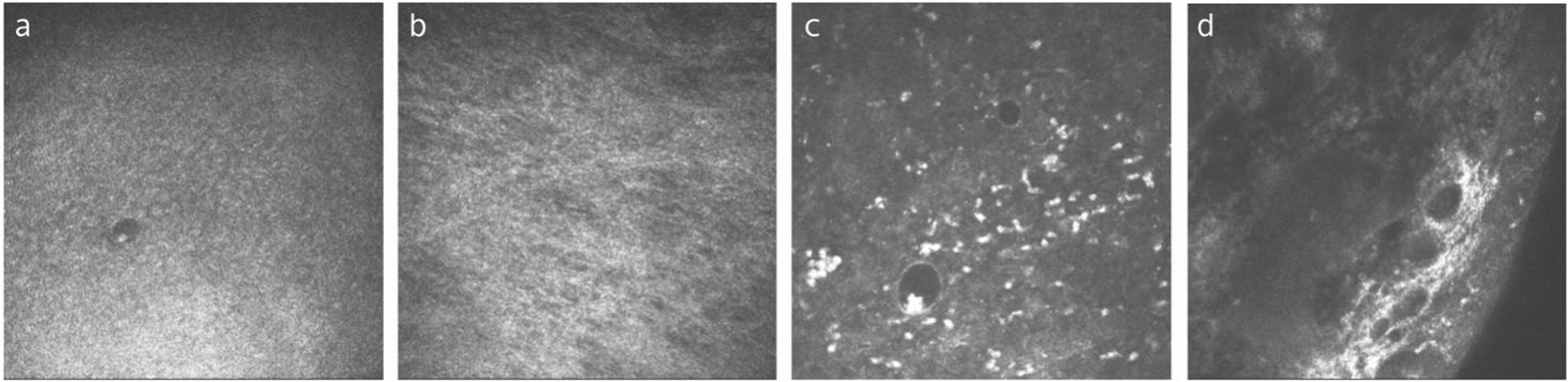
Representative in-vivo confocal microscopy (IVCM) images from a 69-year-old healthy male and a 65-year-old male who underwent vitrectomy for retinal detachment 5 years ago. **a** Microcysts in the conjunctival epithelium manifested as a relatively smaller size with a low microcyst density. **b** The normal reflective intensity of the fiber network in the conjunctival lamina propria. **c** In the surgery-affected area, the epithelial conjunctival microcysts tend to be larger and contain numerous cellular elements (supposedly lymphocytes) surrounding or inside them. **d** The conjunctival lamina propria manifested as numerous clustered large microcysts and a hyperreflective fibrous network

IVCM has also been used to assess the changes in unconventional AH outflow in patients with rhegmatogenous retinal detachment (RRD) after scleral buckling (SB) surgery. Carpineto et al. [140] reported that the MMD and MMA were significantly higher in RRD eyes than in normal eyes, and in RRD-affected quadrants than the unaffected quadrants of the same eyes [140]. The difference between the RRD-affected and normal eyes was consistently maintained after the SB surgery and at the follow-up visit, with both MMD and MMA negatively correlating with IOP [140]. The IVCM findings demonstrate the potential impact of SB surgery on IOP. Figure 9 shows the morphological changes in the conjunctiva following vitrectomy surgery for retinal detachment.

#### Ochronosis

Ochronosis, also called alkaptonuria, is a rare inherited disorder that causes the deposition of dark pigments in collagenous tissues [141]. Although it typically affects the sclera and episclera near the insertion of the recti muscle, rather than the cornea and conjunctiva, ochronosis should be considered in the differential diagnosis of pigmentary lesions [142]. Marked degenerative changes such as vacuoles and fragmentation of collagen fibers were observed in the affected conjunctiva on IVCM scans [143]. Inflammatory cells beneath the conjunctival epithelium and hyperreflective deposits of various shapes in the substantia propria can also be observed [143].

In summary, IVCM is a useful tool to help with the diagnosis and management of various conjunctival diseases. IVCM allows clinicians to assess disease severity and treatment efficacy through various indications. It can evaluate the extent of inflammatory infiltration in DED, determine the activity status or recurrence of pterygium lesions, and assess the function of filtering blebs in glaucoma patients, providing indications of potential filtration failure. Additionally, IVCM-based grading systems can aid in monitoring the progression of trachomatous scarring. IVCM also enables the detection of impacts of topical or systemic medications on the conjunctiva, such as inflammatory responses, loss of GCs, and more severe conditions such as conjunctival fibrotic changes. Therefore, it provides evidence for monitoring the potential ocular surface toxicity of drugs. For individuals with conjunctivitis or keratoconjunctivitis, IVCM serves as a supplementary tool to biopsy-based histopathology for diagnosis. Despite the strong correlation between IVCM and pathology in diagnosing pigmented conjunctival tumors, IVCM should be used primarily for initial diagnosis and differentiation rather than for definitive diagnosis. This recommendation arises due to IVCM's limited reliability in distinguishing between malignant and benign conjunctival lesions, including neoplasms or pterygium.

### Conclusions and future direction

IVCM opens a new avenue for the in-vivo study of conjunctival diseases. This emerging and non-invasive imaging assessment provides an accurate and reproducible assessment of the structural characteristics of the conjunctiva not only under different treatment modalities but also over time as the disease progresses. The growing applications of IVCM enable clinicians to gain a better understanding of the mechanisms of disease at the cellular level and to add value to definitive diagnosis [144]. IVCM can also reveal cellular and tissue alteration following medical therapy and surgical procedures, rendering it an indicative and longitudinal monitoring tool. In addition, it can provide valuable prognostic evidence by assessing filtration function and inflammation status. However, IVCM still has limitations. The resolution of the image can be affected by the depth and the light transmittance of the scanned target. Screening children is challenging, as it is a contact system. For the identification of cell phenotypes, histological examination is still the golden standard. Positioning the everted upper lid and maintaining it in a stable and everted state during the scan can be challenging when examining the tarsal conjunctiva, particularly in patients with trachomatous conjunctival scarring, who often have limited eyelid mobility. Therefore, an additional physician to fix the eyelid may be needed. Future studies with wide-field scanning systems will allow for a larger detection area. Automated measurement using machine learning or deep learning algorithm would increase the efficiency of quantitative conjunctival morphology analysis, facilitating data-rich research and improving clinical practice.

## Data Availability

Not applicable.

## Abbreviations

IVCM: In-vivo confocal microscopy
CALT: Conjunctiva-associated lymphoid tissue
GCs: Goblet cells
DCs: Dendritic cells
DED: Dry eye disease
SSDE: Sjögren’s syndrome dry eye
NSSDE: Non-Sjögren’s syndrome dry eye
IOP: Intraocular pressure
AH: Aqueous humor
POAG: Primary open-angle glaucoma
MMD: Microcysts mean density
MMA: Microcysts mean area
BAK: Benzalkonium chloride
PAM: Primary acquired melanosis
OSSN: Ocular surface squamous neoplasia
MALT: Mucosa-associated lymphoid tissue
VKC: Vernal keratoconjunctivitis
SLK: Superior limbic keratoconjunctivitis
GVHD: Chronic graft-versus-host disease
RRD: Rhegmatogenous retinal detachment
SB: Scleral buckling
